# Genome-wide investigation of the *ZF-HD* gene family in two varieties of alfalfa (*Medicago sativa* L.) and its expression pattern under alkaline stress

**DOI:** 10.1186/s12864-022-08309-x

**Published:** 2022-02-21

**Authors:** Kai He, Chunxin Li, Zhenyue Zhang, Lifeng Zhan, Chunlong Cong, Depeng Zhang, Hua Cai

**Affiliations:** College of Life Sciences, Northeast Agricultural University, Harbin, 150030 China

**Keywords:** *Medicago sativa*, *ZF-HD* gene family, Collinearity analysis, Alkaline stress

## Abstract

**Background:**

Zinc finger homeodomain (ZHD) protein is a plant-specific transcription factor and a potential regulator of phosphoenolpyruvate carboxylase (PEPCase)-coding genes, and it also participates in plant growth regulation and abiotic stress responses. To study the function of *MsZF-HD* genes in the alkaline stress response, this paper assessed biological information and performed transcriptome analysis of the *MsZF-HD* gene family by using the genomes of two different varieties of alfalfa (XinJiangDa Ye and Zhongmu No. 1).

**Results:**

In total, 49 and 11 *MsZF-HD* genes were identified in the two different varieties respectively, including the alleles of XinJiangDa Ye. According to their phylogenetic relationships, the 60 *MsZF-HD* genes were divided into 5 ZHD subfamilies and 1 MIF subfamily. A total of 88.3% of *MsZF-HD* genes do not contain introns and are unevenly distributed among the 6 chromosomes of alfalfa. A collinearity analysis indicated that 26 genes of XinJiangDa Ye have no orthologous genes in Zhongmu No. 1, although these genes (such as *ZHD-X1–2*, *ZHD-X3–2* and *ZHD-X4–2*) have homologous genes in *Arabidopsis thaliana*, *Medicago truncatula* and *Glycine max*. Through RNA-seq and qRT–PCR verification, it was found that *MsZF-HD* genes are downregulated to participate in the alkaline stress response.

**Conclusion:**

The results of this study may lay the foundation for the cloning and functional study of *MsZF-HD* genes and provide a theoretical basis for revealing the difference between XinJiangDa Ye and Zhongmu No. 1 at the genome level.

**Supplementary Information:**

The online version contains supplementary material available at 10.1186/s12864-022-08309-x.

## Introduction

Transcription factors (TFs) play a vital role in the growth and development of plants in response to abiotic stress by promoting the self-regulation and regulation of downstream target gene expression [1, 2]. ZF-HD (zinc finger homeodomain, ZHD) is a plant-specific transcription factor that was first reported in the C_4_ plant *Flaveria trinervia*, and a potential regulatory factor of phosphoenolpyruvate carboxylase (PEPCase)-encoding genes [1]. The amino acid sequences of ZF-HD TFs have a conserved HD domain (homeodomain) that contains 60 amino acids that can be folded into a triple helix structure and specifically bind to the major groove of DNA [3, 4]. In addition to the HD domain, ZF-HD also has a zinc finger structure (ZF domain) which is widely present in a variety of regulatory proteins and can specifically bind to DNA/RNA sequences and participate in protein interactions [5]. Interestingly, there is also a type of MIF (mini zinc finger) protein that is characterized by a very short sequence containing a central zinc finger domain [6]. Unlike the ZHD proteins, MIFs are found only in seed plants and possibly originate from ZHDs by the loss of the homeodomain and subsequent divergence in seed plants. Alternatively, it might be that ZHDs originated from MIFs by gaining the HD domain [7]. Thus, the ZHDs and MIFs should both belong to ZF-HDs, thereby dividing the ZF-HDs into two different groups. Currently, the ZF-HD family has been authenticated in *Arabidopsis thaliana* [2], *Vitis vinifera* [8], *Glycine max* [9], *Solanum lycopersicum* [10], *Fagopyrum tataricum* [11], *Triticum aestivum* [12], etc.

ZF-HD proteins are expressed predominantly or exclusively in floral tissue, indicating a likely regulatory role during floral development [2]. The ZF-HD class of homeodomain proteins may also be involved in the photosynthesis-related mesophyll-specific gene expression of phosphoenolpyruvate carboxylase in C_4_ species [2] and in pathogen signaling and plant defense mechanisms [13]. Additionally, ZF-HD proteins were found to play various important roles in abiotic stress [14]. In *A. thaliana*, 17 *ZF-HD* family genes have been shown to have great significance in the development of flowers [1, 2]. *AtZHD1* is induced by drought, salinity and abscisic acid (ABA), and the overexpression of *ZF-HD1* along with *NAC* genes confers tolerance to drought stress. It was also found that ZF-HD1 binds to the promoter of EARLY RESPONSE TO DEHYDRATION STRESS 1 (ERD1) [8]. Another report showed that *AtMIF1* is overexpressed in *Arabidopsis*, suggesting that *AtMIF1* may be involved in the regulation of plant development by multiple hormones. It is possible that AtMIF1 interacts with ZHD proteins via the ZF domain and its overexpression interferes with the normal functions of ZHD proteins [6]. Two soybean ZF-HD proteins were confirmed to bind with the promoter of the gene encoding calmodulin isoform 4 (*GmCaM4*) and are highly expressed under pathogen inoculation [13]. In rice, 4 ZF-HD proteins were identified that bind to the promoter of *OsDREB1B* (DROUGHT RESPONSE ELEMENT BINDING 1B) and show differential transcriptional levels with *OsDREB1B* under different abiotic stress conditions, which is illustrative of the crosstalk between stress signaling pathways [15]. Additionally, *OsZHD1, 2, 4,* and *8* could form homo and heterodimers, and dimerization may play a prominent transactivation role in the regulation of the *OsDREB1B* response to abiotic stress.

Alfalfa (*Medicago sativa* L.) is the major forage protein source for livestock and has been planted in more than 80 countries around the world, with a planting area of more than 30 million hectares [16]. Due to the autotetraploidy and self-incompatibility of alfalfa, the lack of a reference genome makes artificial improvement very challenging. Although chromosome-level genome sequences based on XinJiangDa Ye [17] and Zhongmu No. 1 [18] have been published, the *ZF-HD* family has not been comprehensively analyzed in alfalfa. Hence, we used the genome of two different varieties of alfalfa to conduct a genome-wide scan and bioinformatics analysis of the *MsZF-HD* genes. The analysis of this article may provide a valuable clue for further revealing the biological functions of *ZF-HD* family genes and the cloning of *MsZF-HD* genes.

## Results

### Identification of *ZF-HD* family genes in *Medicago sativa* L*.*

A total of 49 *MsZF-HD* genes including alleles, in XinJiangDa Ye and 11 genes in Zhongmu No. 1 were identified (only the longest splice variant was reserved at each genomic locus for further analysis). For the 60 putative MsZF-HD proteins, their molecular weight (Mw) and isoelectric point (pI) values were also determined by the Expasy online service (Supplementary Table 1). The protein sequences encoded by the *MsZF-HD* genes ranged in length from 75 amino acids for MsMIF-Z1 to 400 amino acids for MsZHD-X1–13, with an average of approximately 232 amino acids. The predicted Mw of the MsZF-HD proteins ranged from 8.14 to 43.36 kDa, and the theoretical pI values ranged from 5.05 to 9.07. These results confirmed that the 60 MsZF-HD proteins had large differences in sequence and protein characteristics.

### Phylogenetic analysis of *M. sativa ZF-HD* genes

**Fig. 1:**
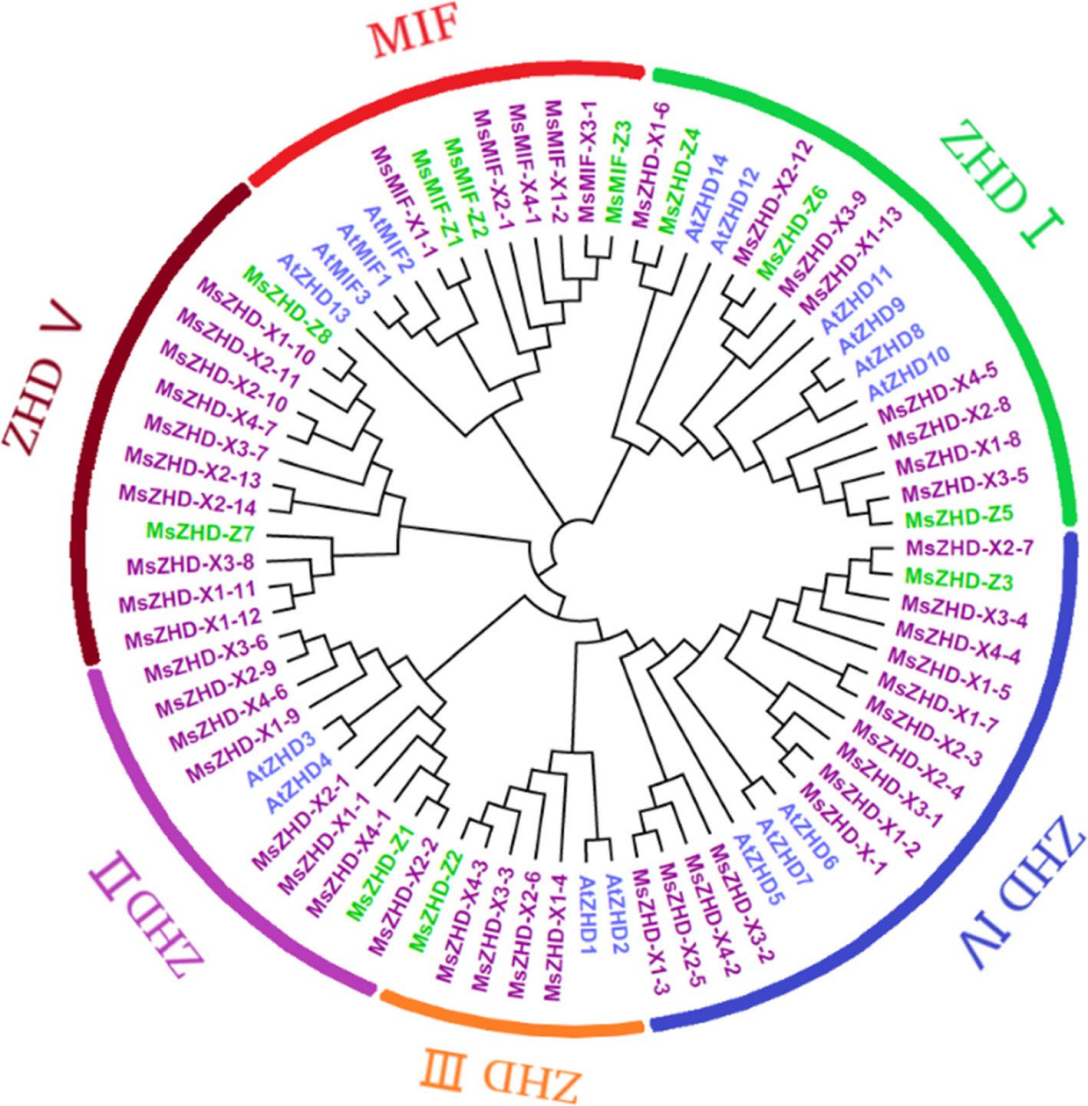
Unrooted phylogenetic tree based on the relationship between *Medicago sativa* L. and *Arabidopsis thaliana*. Colored branches indicate different subfamilies

MEGA X software was used to construct a phylogenetic tree of 60 *M. sativa* and 17 *Arabidopsis* ZF-HDs (Fig. 1). According to the phylogenetic tree, *MsZF-HD* genes can be divided into six subfamilies: ZHD I, ZHD II, ZHD III, ZHD IV, ZHD V and MIF. The number of ZHD IV subfamily proteins is the largest, with a total of 15 *MsZHD*s and *AtZHD5–7*; while the number of ZHD III subfamily proteins is the least, with 5 *MsZHD*s and *AtZHD*1. In addition, 5 XinJiangDa Ye and 3 Zhongmu No.1 MIF proteins were assigned to the MIF subgroup and *AtMIF1–3* were separately classified.

### Conserved motifs and gene structures

**Fig. 2:**
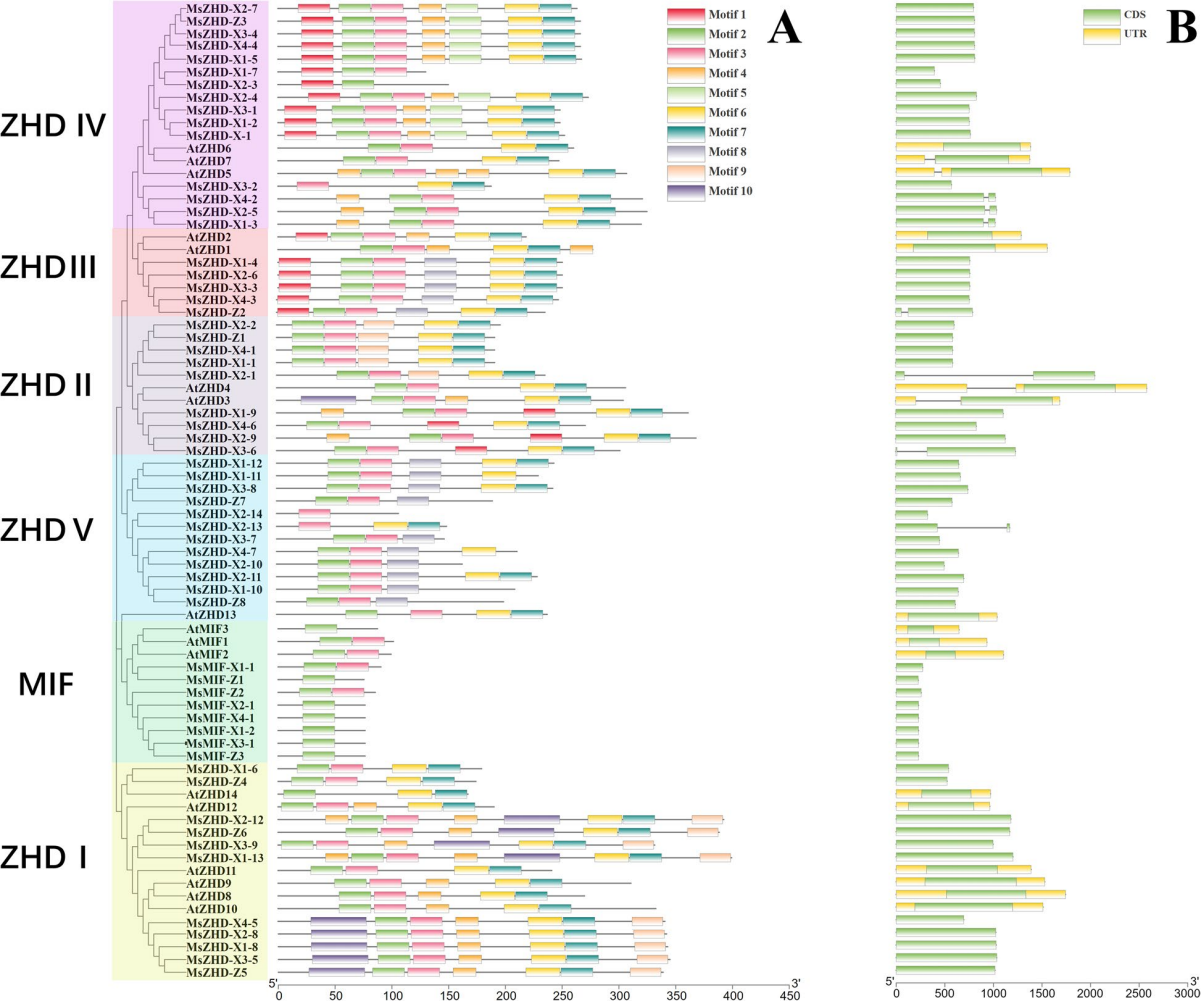
Phylogenetic relationships, gene structures and architectures of the conserved protein motifs. **A** The motif composition of the MsZF-HD proteins. The motifs, numbered 1–10, are displayed in different colored boxes. **B** Exon-intron structures of the *MsZF-HD* genes. Yellow boxes indicate untranslated 5′- and 3′-regions; green boxes indicate exons; and black lines indicate introns

To support the phylogenetic reconstruction, a motif analysis was performed by transferring the 60 MsZF-HD and 17AtZF-HD amino acid sequences to the online MEME Web server (Fig. 2). Ten conserved motifs were identified in the MsZF-HD proteins, and the results were largely consistent with the phylogenetic analysis. Motif 2 and motif 3 were detected in most of the MsZF-HD proteins except for AtZHD-14, MsZHD-X2–3, MsZHD-X3–2, MsZHD-X2–13, MsZHD-X2–14 and 7 MIFs (including only motif 2 or motif 3). Each protein contains an average of 5 motifs, and the MIF subfamily contains only 1 or 2. The motif distribution pattern of MsZF-HD proteins in each subfamily was basically the same, such that ZHD III subfamily members all contained motifs 2, 3, 6, 7 and 8.

To identify the characteristics of the *MsZF-HD* gene family, the structure of these *ZF-HD* genes was analyzed (Fig. 2B). Most *MsZF-HD* genes do not contain introns, and only a few of them contain one intron, such as *MsZHD-X1–3*, *MsZHD-X2–1*, *MsZHD-X2–5*, *MsZHD-X2–13*, *MsZHD-X3–6*, *MsZHD-X4–2* and *MsZHD-Z2*. The results revealed that members of the MIF and ZHD I subfamilies do not contain any introns.

### Chromosome distribution and gene duplication analysis

**Fig. 3:**
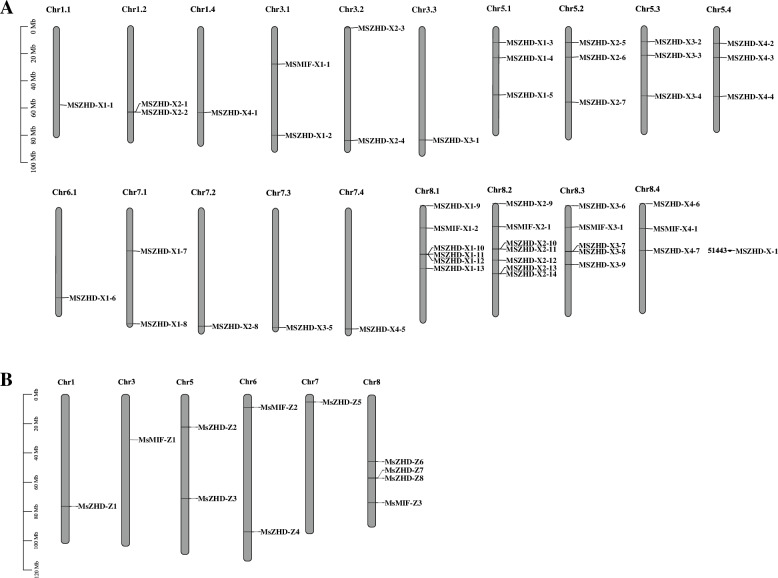
Chromosomal location of *MsZF-HD* genes in *Medicago sativa* L. **A** XinJiangDa Ye. **B** Zhongmu No. 1. Tandem genes are linked by red lines and the chromosomes without *MsZF-HD* genes were omitted

**Fig. 4:**
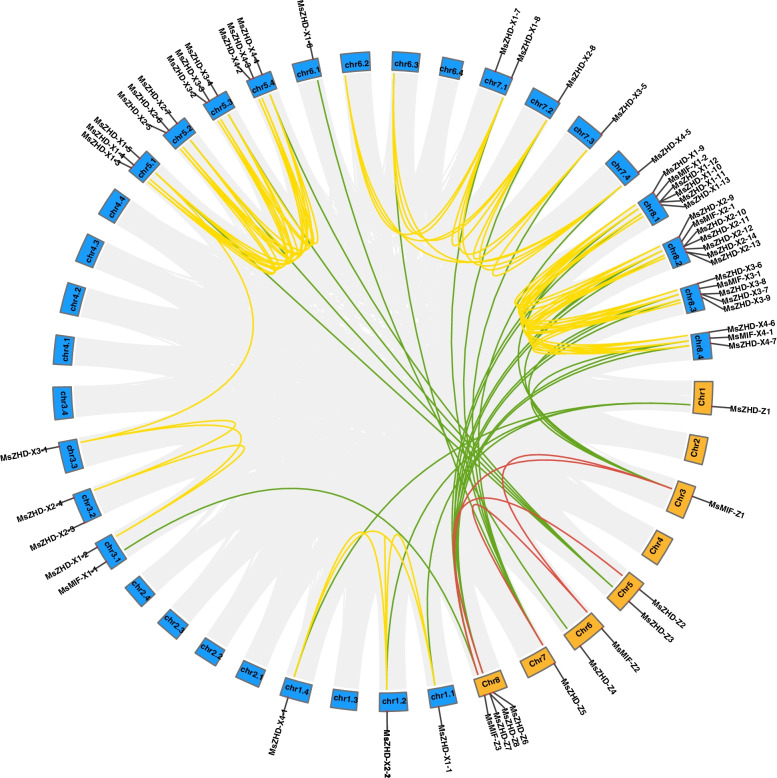
Orthologous genes of *MsZF-HD* genes between XinJiangDa Ye and Zhongmu No. 1. Gray lines in the background indicate the collinear blocks within two different varieties’ genomes; red and yellow lines highlight the syntenic *MsZF-HD* gene pairs in XinJiangDa Ye and Zhongmu No. 1 respectively; green lines highlight the syntenic *MsZF-HD* gene pairs between the two different varieties

The results showed that 60 *MsZF-HD* genes were unevenly distributed on six chromosomes of XinJiangDa Ye and Zhongmu No. 1 while none were distributed on Chr2 and Chr4 (Fig. 3). The locations of *MsZF-HD* genes from the same subfamily were basically the same in XinJiangDa Ye and Zhongmu No. 1. For example, the chromosomes and relative positions of *MsMIF-X1–1* and *MsMIF-Z1* in the two varieties were fixed, indicating that the arrangement of *MsZF-HD* genes was highly conserved. When the query coverage and consistency of the candidate genes were ≥ 80, they were considered repetitive genes. Chromosomal regions within the 200 kb range of two or more genes were defined as tandem replication events [11]. Therefore, the analysis of *MsZF-HD* gene duplication showed that only one tandem repeat gene (*MsZHD-X1–11* and *MsZHD-X1–12*) was found on chromosome 8.1 in XinJiangDa Ye and abundant genes were involved in fragment repeat events (Fig. 4).

**Fig. 5:**
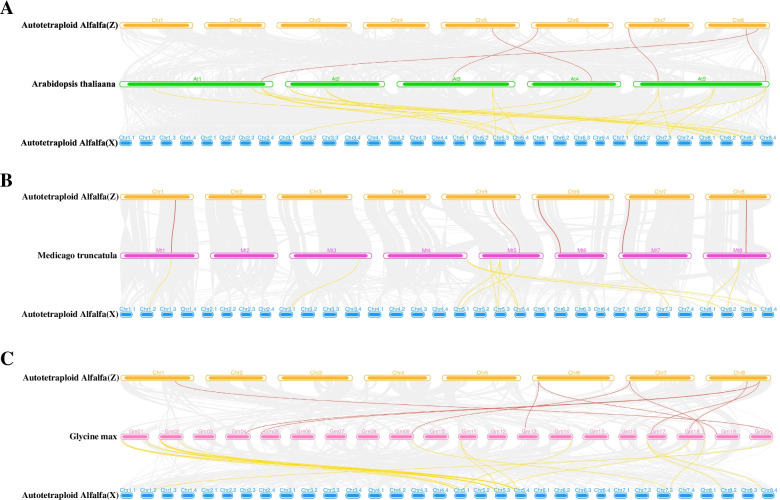
Orthologous genes of *ZF-HD* genes between *Medicago sativa*, *Arabidopsis thaliana*, *Medicago truncatula* and *Glycine max*. Gray lines in the background indicate the collinear blocks within alfalfa and other plant genomes; red and yellow lines highlight the syntenic *MsZF-HD* gene pairs of XinJiangDa Ye and Zhongmu No. 1 respectively

To further infer the phylogenetic relationship between alfalfa and closely related dicotyledonous plants, we analyzed the collinear relationships between alfalfa and the other three plants (Fig. 5). The results showed that 28 *MsZF-HD* genes of XinJiangDa Ye were collinear with *A. thaliana* genes, followed by *G. max* (17) and *M. truncatula* (15), and 7 *MsZF-HD* genes of Zhongmu No. 1 were collinear with *M. truncatula* and *G. max* genes, followed by *A. thaliana* (5). The collinear relationship between the *MsZF-HD* genes of Chr5 and that of the other three plants was significantly greater compared with that of the other chromosomes.

### Promoter region analysis of the *MsZF-HD* genes

**Fig. 6:**
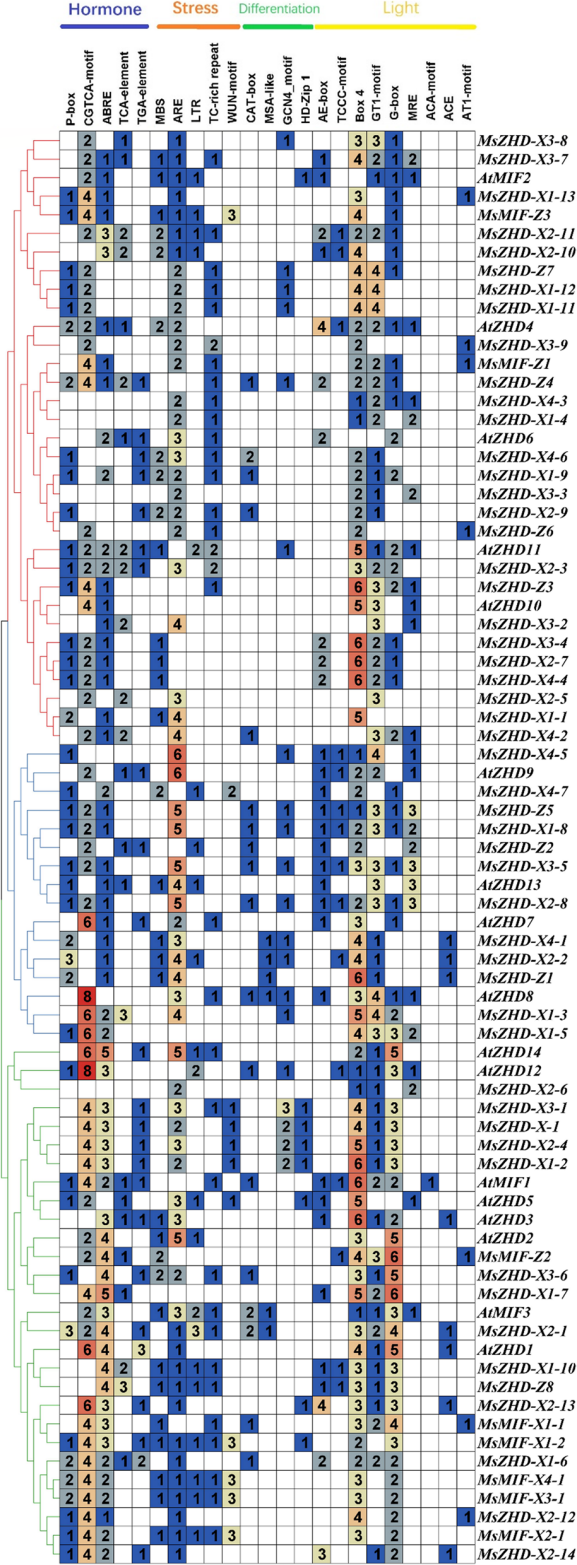
Cis-elements in the promoter region of the *MsZF-HD* genes. The number of cis-elements in the promoter region of each *MsZF-HD* genes (2 kb upstream of the translation start site) were indicated by the different colors and numbers in the grid

The identification of cis-regulatory elements in the promoter region can provide references for tissue-specific or stress-response expression patterns of genes. The 2.0-kb promoter region located upstream of the transcriptional start site (ATG) of each *MsZF-HD* gene was analyzed to determine their potential regulatory mechanisms by using online Plant CARE software. Several light response, hormone response and stress response elements were relatively highly abundant among these cis-elements (Fig. 6). The cis-elements of the *MsZF-HD* genes belonging to the same subfamily did not show the same pattern. Hormone-related cis-elements are predominantly represented by the MeJA (CGTCA-motif) and ABA (ABRE) response elements, which are endogenous growth regulators of higher plants and have physiological effects, such as inhibiting plant growth and germination, promoting senescence, and improving resistance (Fujita et al. 2013). These elements include 5 ABREs in *MsZHD-X1–7* and 6 CGTCA motifs in *MsZHD-X1–3*, *MsZHD-X1–5*, and *MsZHD-X2–13*. Stress response elements can be divided into five categories: MBS, ARE, LTR, TC-rich repeats and WUN-motif. Among them, AREs (anaerobic response elements) were the most abundant, *MsZHD-X4–5* and *AtZHD9* all contained 6 AREs, and almost 80% of the upstream *MsZF-HD* genes contained ARE elements. In addition, Box-4, GT1 motif, and G-box that respond to light were also prominent in the upstream of *MsZF-HD* genes, especially Box-4. 90baifenzhi of the upstream of *MsZF-HD* genes contained BOX-4 element, and the number of these motifs was also the largest compared with the other motifs.

### Expression of *MsZF-HD* genes in response to abiotic treatment

**Fig. 7:**
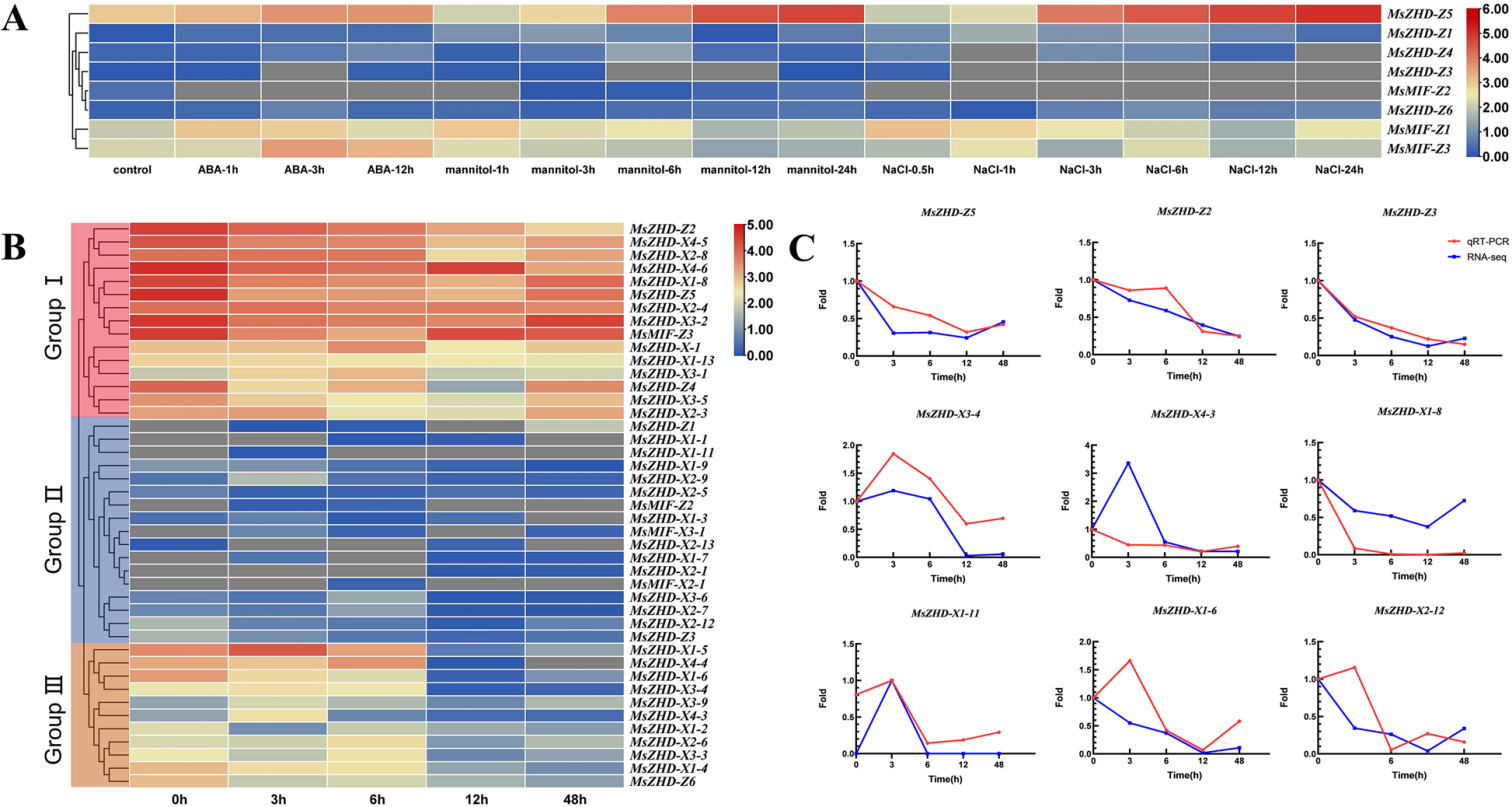
Expression of *MsZF-HD* genes in response to abiotic treatment. **A** The express patern of *MsZF-HD* genes under 250 mM NaCl, 10 M ABA and 400 mM mannitol treatment. The FPKMs were calculated for expression values from RNA-Seq data. **B** The *MsZF-HD* genes were clustered into three groups basing on their expressional profiles under alkaline stress. **C** qRT-PCR verification of the RNA-seq result

To determine whether *MsZF-HD* genes respond to abiotic stress, the transcriptome data of Zhongmu No. 1 under different abiotic treatments (250 mM NaCl, 10 μM ABA and 400 mM mannitol) were obtained from the NCBI and assembled into the genome of Zhongmu No.1 (Additional file 1: Table S2) to investigate *MsZF-HD* genes in response to abiotic stress. The expression levels of these genes were presented in a heatmap (Fig. 7A). Of the 11 *MsZF-HD* genes in Zhongmu No. 1, the transcript levels (FPKM values) of three genes indicated that they were not expressed in any tissue while the remaining 8 genes were expressed at least once. Among them, most *MsZF-HD* genes were differentially expressed in all the examined treatments. For example, *MsZHD-Z5* showed a tendency of upregulation under all three treatments, while most genes (such as *MsZHD-Z*1, *MsZHD-Z*4, and *MsMIF*-Z6) showed a low expression level. The expression of *MsMIF-Z3* was upregulated under the ABA treatment, while the expression patterns under the mannitol and NaCl treatments were the opposite.

We also performed RNA-seq to detect the expression levels of the *MsZF-HD* genes under alkaline stress (Fig. 7B). After mapping those reads to the alfalfa genome, we determined the expression of 43 *MsZF-HD* genes that were expressed in at least one time period in the RNA-Seq data. All *MsZF-HD* genes decreased with time and could be clustered into three groups based on the expression profiles. Group I included genes with high expression level, and members of subfamily I occupied the main position, followed by ZHD IV. Almost all genes in subfamily V were not expressed, and the expression level of only two expressed genes (*MsZHD-X1–11* and *MsZHD-X2–13*) belonging to group II was extremely low. ZHD II and III were almost all concentrated in group III, with low expression level. To further confirm the RNA-seq result of these *MsZF-HD* genes, qRT–PCR was performed for the *MsZF-HD* genes from different clusters (Fig. 7C). The expression patterns of most *MsZF-HD* genes in the qRT–PCR analysis were consistent with the RNA-Seq analysis, implying that our RNA-seq results are highly reliable. The expression level of most *ZF-HD* genes were very low, and the *ZF-HD* genes were downregulated after the alkaline treatment from 3 h to 48 h. The *MsZF-HD* genes from the same subfamily also have different expression patterns, indicating that these genes respond to alkali stress in different levels. All the *MsZF-HD* genes were decreased under alkaline stress, which suggested that the response of these genes may be negatively regulated by abiotic stress.

## Discussion

With the rapid development of high-throughput sequencing technologies, the *ZF-HD* gene family has been studied in many model plants, such as *A. thaliana* (17), *G. max* (36), *T. aestivum* (37), *F. tataricum* (20), *V. vinifera* (13), *S. lycopersicum* (22), *Gossypium hirsutum* (35), *Brassica napus* (62), etc. In this study, 49 and 11 *MsZF-HD* genes were identified in XinJiangDa Ye and Zhongmu No. 1 by scanning the whole genome of two varieties of alfalfa, respectively. In XinjiangDa Ye, the number of *MsZF-HD* genes was significantly more than that of Arabidopsis and other plants. That was mainly due to the fact that the genome of XinJiangDa Ye is an allele-aware chromosome-level genome consisting of 32 allelic chromosomes. In hence, *MsZF-HD* genes identified include a portion of allelic genes. In our judgment, there are 10 pairs of allelic genes which can be clearly observed in Fig. 3. However, among the four nonhomologous chromosome groups of XinJiangDa Ye, the largest group was the second with 15 *MsZF-HD* genes, which is significantly more than that of Zhongmu No. 1. The number of *MsZF-HD* genes in XinJiangDa Ye was much more than that of Zhongmu No. 1, which can lead to the formation of a flexible combinatorial system of transcription factors and may allow for subtle adjustments to many different environmental conditions because of the different binding to cis-elements and flexible dimerization [19]. In addition, different dimers have different reaction conditions for protein binding response [20].

To study the developmental and evolutionary relationship between *M. sativa* and *Arabidopsis*, a phylogenetic tree was constructed using all ZF-HD amino acid sequences of XinJiangDa Ye, Zhongmu No. 1 and *Arabidopsis*. The branches on these phylogenetic tree branches were similar to those in a previous *Arabidopsis* report, which means that most ZF-HD proteins have homology and ZF-HD proteins are relatively conserved among different plants [10]. Although there is a relatively close evolutionary distance between alfalfa and *M. truncatula*, the number of *MsZF-HD* gene homologous genes in *M. truncatula* is not greater than that in *G. max*. This finding may be related to the greater number of gene duplication events in *G. max*, which leads to more *ZF-HD* genes. Some *MsZF-HD* genes are related to at least two pairs of homologous genes, such as *MsZHD-X3–2*, *MsZHD-X3–5* and *MsZHD-Z7*, which may play an important role in the evolution of the *MsZF-HD* family. In addition, there are 26 *MsZF-HD* genes in XinJiangDa Ye that do not have orthologous genes in Zhongmu No. 1. However, those genes specific to XinJiangDa Ye (such as *MsZHD-X1–2, MsZHD-X1–3*, and *MsZHD-X1–13*) have homologous genes in the genomes of other dicotyledonous plants. It was proposed that increases or decreases in the genes on a single chromosome in the karyotype have a greater impact on the phenotype than changes in the whole genome [21]. Therefore, differences between the genomes of XinJiangDa Ye and Zhongmu No. 1 do exist, and the difference may have certain significance to excavate molecular marker genes in later alfalfa molecular breeding. There are a large number of collinearity relationships between nonhomologous chromosomes of alfalfa, and only a pair of tandem repeat genes were found in XinJiangDa Ye, indicating that the fragment repeats played an important role in the expansion of *MsZF-HD* genes. For example, *Arabidopsis thaliana* experienced αand βduplication in evolution which led to the expansion of gene family members [22]. Tandem duplication of *MsZF-HD* genes were not observed in Zhongmu No. 1, which is consistent with the fewer *MsZF-HD* genes in Zhongmu No. 1, thus indicating that there were more active gene duplication events in XinJiangDa Ye.

In general, TFs always harbor some important conserved domains and motifs for their regulatory function [23]. Through the MEME suite, 10 motifs were identified in MsZF-HD proteins. The distribution of conserved motifs in the same subfamily was basically consistent, and each conserved motif have its own unique function. Almost all ZF-HD proteins contain motif 2 and motif 3, while the *MsZHD-X2–3*, *MsZHD-X3–2*, *MsZHD-X2–13*, *MsZHD-X2–14* and 6 MIF genes do not contain motif 2 or 3. A SMART online analysis showed that motif 2 and motif 3 were in the ZF-HD_dimer domain and indicated that they may be involved in the formation of homo or heterodimers and a zinc finger [1]. These results indicate that the ZF-HD_dimer domain is highly conserved in ZF-HD proteins and plays an important role in binding DNA. There are three main mechanisms for changes in the structure of gene exons and introns (gain or loss, exonization or pseudoexonization, and insertion and deletion of exons or introns), which each lead to differences in gene structure [24]. When genes have no introns, they are not easy to connect and their functions are relatively conservative [25]. Almost all *MsZF-HD* genes have no introns, revealing that they play an important role in the growth and development of alfalfa. This result is consistent with a previously reported result showing that the *ZF-HD* genes have almost no introns [1]. Introns of the few *MsZF-HD* genes may be obtained by mutations during inheritance or evolution. The number of genes containing introns in XinJiangDa Ye is greater than that of Zhongmu No. 1, and whether the appearance of introns in these genes affects the original functions is a question that needs further research.

*ZF-HD* genes are differentially expressed in different tissues of different species, indicating that they have an important role in the development of plant tissues [10]. ZF-HD TFs were first identified as a potential regulator of the PEPCase gene in the C_4_ plant *Flaveria bidentis*, and they are involved in a variety of biological processes, such as the response to abiotic stress [15] and hormones, during plant development [2]. ZF-HD TFs have been shown to have function in large complexes during the period in the early stages of flower development [26], fruit development [11], salt stress [27] and drought stress [28]. However, the expression of *ZF-HD* genes under alkaline stress has not been previously investigated. Therefore, both XinJiangDa Ye and Zhongmu No. 1 were treated with alkaline stress and samples were taken at 5 different time periods to explore the expression of *MsZF-HD* genes in response to alkaline stress. Many *MsZF-HD* genes could not be detected after the alkaline treatment, which may indicate that these genes are expressed only in specific environmental conditions or tissues. It was worth noting that the differentially expressed gene in the root tips under salt stress were not the same as those in the leaves under alkali stress. For example, *MsZHD-Z5* was significantly up-regulated in the root tips under salt stress, while down regulated in the leaves under alkali stress. In conclusion, the functions of *MsZF-HD* gene are different under salt and alkali stress. Most *MsZF-HD* genes expressed the highest levels in the early stage of the alkaline stress treatment and stabilized after 12 h, reaching a very low level, thus indicating that the ZF-HD TFs respond to alkaline stress quickly and may be upstream of the signal transduction pathway. Constitutive overexpression of the *AtMIF1* gene leads to significant developmental defects, including dwarfing, reduced apical dominance, altered flower morphology, reduced hypocotyl length, reduced root growth, and ectopic root hairs on hypocotyls and cotyledons [8]. Similar to the other *ZF-HD* genes, *MsMIF* also included a few genes that could respond to alkaline stress, although there were certain differences. The MIF subfamily genes of alfalfa have a low expression level, and only two expressed genes (*MsMIF-X2–1* and *MsMIF-X3–1*) showed a trend of initially increasing and then decreasing.

## Conclusion

In summary, this study identified 60 *MsZF-HD* genes in *M. sativa* L and then characterized these genes and explored their phylogenetic relationships. The expression analysis showed that most *MsZF-HD*s also respond to alkaline stress, in addition to drought, salt and ABA stress.

## Materials and methods

### Identification and characterization of the ZF-HD family in alfalfa

The genome and annotation information of alfalfa (XinJiangDa Ye and Zhongmu No. 1) were downloaded from the figshare data repository (https://figshare.com). The *A. thaliana* genome and annotation information were downloaded from the TAIR database (https://www.arabidopsis.org/), and the 17 AtZF-HD protein sequences were downloaded from TFDB (http://planttfdb.gao-lab.org/, V5.0). The *MsZF-HD* genes were searched using 2 methods: First, the published AtZF-HD protein sequences were used as probes for a BLAST search against the proteome of alfalfa to obtain candidate *ZF-HD* family members. Second, the hidden Markov model (HMM) profiles of the ZF-HD_dimer domain (PF04770) were downloaded from the Pfam database (http://pfam.xfam.org/) to search for the ZF-HD_dimer domain from the alfalfa proteome using an e-value cut off of 1.0. Duplicate genes obtained by the two methods were removed to obtain the final *MsZF-HD* genes. The NCBI Batch CD-Search (https://www.ncbi.nlm.nih.gov) was utilized to analyze the domains of candidate ZF-HD proteins to ascertain the candidate genes for subsequent analysis. Finally, the nucleotide and deduced amino acid sequences of 60 *MsZF-HD* genes were confirmed for further analysis. The amino acid sequence lengths, molecular weights (MW) and pI values of all predicted MsZF-HD proteins were then determined by ExPASy (http://web.expasy.org/compute_pi/). All related information of *MsZF-HD* genes are listed in Table 1.

**Table 1:** Related information of *MsZF-HD* genes

Gene name	Gene locus	Chromosome location	Length (aa)	PI	Molecular weight (Da)
*MsMIF-Z1*	*MsG0380013130*	chr3:30962255-30,962,028	75	8.97	8141.20
*MsMIF-Z2*	*MsG0680030765*	chr6:8778200-8,778,457	85	8.88	9361.44
*MsMIF-Z3*	*MsG0880046457*	chr8:73611430-73,611,660	76	9.04	8305.48
*MsZHD-Z1*	*MsG0180004327*	chr1:76449042-76,448,458	194	9.16	22,425.74
*MsZHD-Z2*	*MsG0580025668*	chr5:22328164-22,327,376	239	9.14	25,973.01
*MsZHD-Z3*	*MsG0580027925*	chr5:71184035-71,184,838	267	6.46	29,104.05
*MsZHD-Z4*	*MsG0680034750*	chr6:93921235-93,921,759	174	8.48	19,211.30
*MsZHD-Z5*	*MsG0780036244*	chr7:5192044-5,191,028	338	8.20	37,839.15
*MsZHD-Z6*	*MsG0880044604*	chr8:45574688-45,575,857	389	9.06	42,303.86
*MsZHD-Z7*	*MsG0880045269*	chr8:56721743-56,721,165	192	7.08	21,509.13
*MsZHD-Z8*	*MsG0880045282*	chr8:56879882-56,880,490	202	7.65	22,289.51
*MsZHD-X-1*	*MS.gene020409*	scaffold51443:180617-181,378	253	9.12	27,014.18
*MsMIF-X1-1*	*MS.gene018941*	chr3.1:27683365-27,683,093	90	9.04	10,019.31
*MsMIF-X1-2*	*MS.gene061719*	chr8.1:16702902-16,702,672	76	9.04	8305.48
*MsMIF-X2-1*	*MS.gene033123*	chr8.2:17333400-17,333,170	76	9.04	8305.48
*MsMIF-X3-1*	*MS.gene58432*	chr8.3:15992545-15,992,775	76	9.02	8265.41
*MsMIF-X4-1*	*MS.gene044382*	chr8.4:18692730-18,692,500	76	9.02	8265.41
*MsZHD-X1-1*	*MS.gene062215*	chr1.1:57718340-57,717,756	194	8.90	22,419.74
*MsZHD-X1-2*	*MS.gene48438*	chr3.1:80006686-80,005,937	249	9.12	26,669.81
*MsZHD-X1-3*	*MS.gene047789*	chr5.1:11880941-11,881,956	321	9.21	36,190.73
*MsZHD-X1-4*	*MS.gene86236*	chr5.1:23120941-23,120,186	251	9.23	27,137.36
*MsZHD-X1-5*	*MS.gene64101*	chr5.1:50377119-50,377,925	268	6.46	29,161.10
*MsZHD-X1-6*	*MS.gene000242*	chr6.1:65677061-65,677,600	179	8.16	19,654.82
*MsZHD-X1-7*	*MS.gene98674*	chr7.1:31663989-31,663,597	130	4.72	14,239.10
*MsZHD-X1-8*	*MS.gene91521*	chr7.1:85201850-85,202,878	342	8.20	38,287.68
*MsZHD-X1-9*	*MS.gene012789*	chr8.1:270138-269,035	367	7.56	41,336.66
*MsZHD-X1-10*	*MS.gene008887*	chr8.1:35893129-35,892,491	212	7.65	23,365.85
*MsZHD-X1-11*	*MS.gene46290*	chr8.1:36080136-36,080,799	233	5.81	26,133.83
*MsZHD-X1-12*	*MS.gene008898*	chr8.1:36089558-36,090,205	247	6.18	27,857.81
*MsZHD-X1-13*	*MS.gene34663*	chr8.1:46373743-46,372,541	400	9.06	43,368.84
*MsZHD-X2-1*	*MS.gene88817*	chr1.2:63519845-63,521,889	239	8.92	27,243.13
*MsZHD-X2-2*	*MS.gene07447*	chr1.2:63618407-63,617,808	199	9.14	22,835.08
*MsZHD-X2-3*	*MS.gene53939*	chr3.2:1099072-1,098,620	150	5.09	16,946.49
*MsZHD-X2-4*	*MS.gene052178*	chr3.2:83875635-83,874,811	274	6.08	29,683.68
*MsZHD-X2-5*	*MS.gene010515*	chr5.2:11751825-11,752,855	326	9.21	36,726.33
*MsZHD-X2-6*	*MS.gene027744*	chr5.2:22593459-22,592,704	251	9.23	27,137.36
*MsZHD-X2-7*	*MS.gene010125*	chr5.2:55657198-55,657,992	264	6.76	28,717.69
*MsZHD-X2-8*	*MS.gene043241*	chr7.2:87132177-87,133,202	341	8.20	38,213.59
*MsZHD-X2-9*	*MS.gene95053*	chr8.2:261015 -259,891	374	7.32	42,099.57
*MsZHD-X2-10*	*MS.gene056421*	chr8.2:33683282-33,682,785	165	5.15	17,608.69
*MsZHD-X2-11*	*MS.gene056417*	chr8.2:33735099-33,734,401	232	8.10	25,773.17
*MsZHD-X2-12*	*MS.gene35938*	chr8.2:41933556-41,932,375	393	9.06	42,675.27
*MsZHD-X2-13*	*MS.gene035434*	chr8.2:51873360-51,872,190	151	6.65	17,317.73
*MsZHD-X2-14*	*MS.gene035426*	chr8.2:51974569-51,974,243	108	5.46	11,973.50
*MsZHD-X3-1*	*MS.gene013045*	chr3.3:83536735-83,535,986	249	9.12	26,669.81
*MsZHD-X3-2*	*MS.gene02448*	chr5.3:11832602-11,833,168	188	9.30	21,537.37
*MsZHD-X3-3*	*MS.gene29146*	chr5.3:21737080-21,736,325	251	9.23	27,137.36
*MsZHD-X3-4*	*MS.gene061947*	chr5.3:51674272-51,675,075	267	6.46	29,104.05
*MsZHD-X3-5*	*MS.gene79601*	chr7.3:87786119-87,787,153	344	8.20	38,534.88
*MsZHD-X3-6*	*MS.gene042050*	chr8.3:206351-205,122	306	8.21	34,618.71
*MsZHD-X3-7*	*MS.gene067921*	chr8.3:33702409-33,701,960	149	5.54	16,220.24
*MsZHD-X3-8*	*MS.gene067910*	chr8.3:33916426-33,917,166	246	6.31	27,736.74
*MsZHD-X3-9*	*MS.gene045953*	chr8.3:43574252-43,573,254	332	8.86	36,169.03
*MsZHD-X4-1*	*MS.gene004926*	chr1.4:63200375-63,199,791	194	8.91	22,386.59
*MsZHD-X4-2*	*MS.gene59378*	chr5.4:12454799-12,455,817	322	9.21	36,291.88
*MsZHD-X4-3*	*MS.gene44274*	chr5.4:22839634-22,838,879	251	9.23	27,137.36
*MsZHD-X4-4*	*MS.gene050020*	chr5.4:51440438-51,441,241	267	6.46	29,150.13
*MsZHD-X4-5*	*MS.gene021696*	chr7.4:88978290-88,978,985	341	8.20	38,163.53
*MsZHD-X4-6*	*MS.gene43476*	chr8.4:277389-276,562	275	8.69	31,307.94
*MsZHD-X4-7*	*MS.gene048325*	chr8.4:34663911-34,664,555	214	6.45	23,864.96

### Phylogenetic analysis of ZF-HD proteins

To study the phylogenetic relationships of the different ZF-HDs, the full-length amino acid sequences of ZF-HD members of alfalfa and *Arabidopsis* were used to construct phylogenetic trees by the maximum likelihood (ML) method with 1000 bootstrap replicates through MEGA X software. The parameters were set as follows: protein model: JTT + G + I; gap/missing date treatment: partial deletion; and site coverage cut off: 95%.

### Conserved motif, gene structure, and cis-element analysis of the *ZF-HD* gene family in alfalfa

Conserved motifs and domains of *ZF-HD* genes were predicted by MEME (https://meme-suite.org/meme/tools/meme). The optimized parameters of MEME were as follows: the optimum width of each motif ranged from 6 to 50, and the maximum number of motifs to find was 10. In addition, TBtools [29] was used to display the gene structure and identify the exon/intron boundaries. The Plant CARE database (http://bioinformatics.psb.ugent.be/webtools/plantcare/html/) was used to predict and analyze the cis-elements of 2000 bp upstream sequences of each *MsZF-HD* gene.

### Chromosomal localization and collinearity analysis

To analyze the distribution of *MsZF-HD* genes in alfalfa chromosomes, reference information for the alfalfa genome was obtained. The chromosomal distribution and synteny were analyzed using TBtools. The proteomes of *M. sativa*, *A. thaliana*, *M. truncatula*, and *G. max* were blasted to find duplicated genes between different species. Multiple collinear scanning toolkits (MCScanX) were used to analyze the replication events of the *MsZF-HD* genes. The syntenic relationship between the *MsZF-HD* genes and *ZF-HD* genes from selected plants was determined using Dual Systeny Plotter software (https://github.com/CJ-Chen/TBtools).

### Naming of *ZF-HD* genes

We considered a consistent naming pattern for all *MsZF-HD* genes, with the phylogenetic relationships as well as their subgenome location in XinJiangDa Ye (1, 2, 3 or 4) taken into account. Each gene name starts with an abbreviation for the species, e.g., Ms. for *M. sativa* L., followed by the name of the subfamily, e.g., ‘ZHD’ for ‘zinc finger motif-associated HD’ and ‘MIF’ for ‘mini zinc finger’. In addition, Z and X indicate which variety they represent. The *MsZF-HD* genes belonging to XinJiangDa Ye but on different homologous chromosomes were consecutively numbered and separated by a dash. Hence, the name of the gene with the ID *MS.gene062215* is *MsZHD-X1–1*, indicating that it is a ZHD subfamily gene and the first homologous chromosome and first gene of XinJiangDa Ye.

### Plant material and stress treatment

*M. sativa* (XinJiangDa Ye and Zhongmu No. 1) was employed for the alkaline treatment experiments. Uniform seedlings were transplanted into plastic cylindrical pots (10.5 cm diameter × 9.5 cm high with a 5 mm diameter hole at the bottom, three plants per pot) containing vermiculite and perlite (1:1) under 16/8 h light/dark conditions, 30 °C/25 °C day/night temperatures and 55% relative humidity. The pots were placed in rectangular plastic trays (55 cm × 75 cm, 24 pots per tray). During plant growth, 1/5 Hoagland nutrient solution was applied to the plants once every 2 days. Two-month-old seedlings were utilized for the alkaline treatment. For alkaline stress, plants were treated with 250 mM NaHCO_3_ (pH = 8.5), and leaves of the seedlings were harvested at 0, 3, 6, 24, and 48 h after treatment. Immediately, the collected samples were frozen and stored at − 80 °C until use. For all the above samples, three biological replicates were employed for each sample.

### *Medicago sativa* RNA-seq data analysis

*M. sativa* transcriptome data under different abiotic stresses were downloaded from the NCBI SRA database (http://www.ncbi.nlm.nih.gov, accession numbers SRX4079528–SRX4079572). Different time points of three abiotic stresses, including 250 mM NaCl, 10 μM ABA, and 400 mM mannitol, were analyzed. The transcript abundance is expressed based on the fragments per kilobase of exon model per million mapped reads (FPKM) values. Heat maps for *MsZF-HD* genes that have positive FPKM values in at least one or more of the samples were generated, and the values are shown with log2 (1 + RPKM). We also performed RNA-seq to detect the expression levels of *MsZF-HD* genes under alkaline stress. Clean reads from 30 samples were mapped to the *M. sativa* genome sequencing using SamTool [30]. TopHat and Cufflinks were used to analyze the FPKM results [31]. The FPKM values for *ZF-HD* genes were utilized to generate the heatmap and k-means clustering using R (software) [32].

### RNA extraction and qRT–PCR verification

Total RNA was extracted from each sample using the Plant RNAprep Pure Kit (Kangwei, Beijing China). First-strand cDNA was synthesized from 1 μg of total RNA using a HiScript II Q Select reverse transcriptase kit (Vazyme Biotech, Nanjing, China) according to the manufacturer’s instruction. Gene-specific primers were designed using Primer Premier 5 software. In each reaction, the *GAPDH* gene (accession: XM_003601780.1) was used as an internal reference gene. The relative gene expression levels were calculated according to the 2^−∆∆C(t)^ method. SPSS 2.5 and Excel software were used for data analysis, and Duncan’s method was used to compare significant differences between treatments (*p* < 0.05), which are marked with different lowercase letters. All data were the mean ± standard error of 3 biological replicates with 3 technical replicates. All qRT-PCR validation primers used in the present study are listed in Table 2.

**Table 2:** List of qRT-PCR validation primers used in the present study

Gene ID	Forward primer (5′ - 3′)	Reverse primer (5′ - 3′)
*MsZHD-X1-6*	CGGTAAACAGCCTCCAATG	CCTAACTTCTCAGCAAATCCC
*MsZHD-X1-8*	AACACTTTAGCCAAACGAGA	GATGAACCATTAGCACGAAC
*MsZHD-X1-11*	GGAGGCTACGCTCTTGATG	AGTCGGCTGAGGGAAACG
*MsZHD-X2-12*	TCCCTCAAATGTGCTGCTT	GTGGTGGTTGTGGTGGTG
*MsZHD-X3-4*	GACGGTTGTTGCGAGTTTAT	CAACGGTCTGTGATGTGCC
*MsZHD-X4-3*	TTGATGGTTGCGGTGAGT	GTGGTGGATGGTGATGATGT
*MsZHD-Z2*	TTGATGGTTGCGGTGAGT	GTGGTGGATGGTGATGATGT
*MsZHD-Z3*	GACGGTTGTTGCGAGTTTAT	CAACGGTCTGTGATGTGCC
*MsZHD-Z5*	AACACTTTAGCCAAACGAGA	GATGAACCATTAGCACGAAC
*GAPDH*	GGCTGCATCAAGGAGGAAT	TCCAAGCTCAGCCTCATCAAG

## Supplementary Information

**Additional file 1: Supplementary Table 1.** One-to-one orthologous relationships between *Medicago sativa L.* and other three plant species. **Supplementary Table 2.** The FPKM value of MsZF-HD genes under different abiotic treatment.

## Data Availability

Not applicable.

## References

[1] Windhövel A, Hein I, Dabrowa R, Stockhaus J (2001). Characterization of a novel class of plant homeodomain proteins that bind to the C_4_ phosphoenolpyruvate carboxylase gene of *Flaveria trinervia*. Plant Mol Biol.

[2] Tan QK, Irish VF (2006). The Arabidopsis zinc finger-homeodomain genes encode proteins with unique biochemical properties that are coordinately expressed during floral development. Plant Physiol.

[3] Gehring WJ, Affolter M, Bürglin T (1994). Homeodomain proteins. Annu Rev Biochem.

[4] Wolberger C (1996). Homeodomain interactions. Curr Opin Struct Biol.

[5] Takatsuji H (1999). Zinc-finger proteins: the classical zinc finger emerges in contemporary plant science. Plant Mol Biol.

[6] Hu W, Ma H (2006). Characterization of a novel putative zinc finger gene *MIF1*: involvement in multiple hormonal regulation of Arabidopsis development. Plant J.

[7] Hu W, dePamphilis CW, Ma H (2008). Phylogenetic analysis of the plant-specific zinc finger-homeobox and mini zinc finger gene families. J Integr Plant Biol.

[8] Wang H, Yin X, Li X (2014). Genome-wide identification, evolution and expression analysis of the grape (*Vitis vinifera* L.) zinc finger-homeodomain gene family. Int J Mol Sci.

[9] Bhattacharjee A, Ghangal R, Garg R, Jain M (2015). Genome-wide analysis of homeobox gene family in legumes: identification, gene duplication and expression profiling. PLoS One.

[10] Khatun K, Nath UK, Robin AHK (2017). Genome-wide analysis and expression profiling of zinc finger homeodomain (*ZHD*) family genes reveal likely roles in organ development and stress responses in tomato. BMC Genomics.

[11] Liu M, Sun W, Ma Z (2019). Genome-wide investigation of the *AP2*/*ERF* gene family in tartary buckwheat (*Fagopyum Tataricum*). BMC Plant Biol.

[12] Liu H, Yang Y, Zhang L (2021). Zinc finger-homeodomain transcriptional factors (ZF-HDs) in wheat (*Triticum aestivum* L.): identification, evolution, expression analysis and response to abiotic stresses. Plants (Basel).

[13] Park HC, Kim ML, Lee SM (2007). Pathogen-induced binding of the soybean zinc finger homeodomain proteins *GmZF-HD1* and *GmZF-HD2* to two repeats of ATTA homeodomain binding site in the calmodulin isoform 4 (*GmCaM4*) promoter. Nucleic Acids Res.

[14] Shalmani A, Muhammad I, Sharif R (2019). Zinc finger-homeodomain genes: evolution, functional differentiation, and expression profiling under flowering-related treatments and abiotic stresses in plants. Evol Bioinform Online.

[15] Figueiredo DD, Barros PM, Cordeiro AM (2012). Seven zinc-finger transcription factors are novel regulators of the stress responsive gene *OsDREB1B*. J Exp Bot.

[16] Mielmann A (2013). The utilisation of lucerne (*Medicago sativa*): a review. Br Food J.

[17] Chen H, Zeng Y, Yang Y (2020). Allele-aware chromosome-level genome assembly and efficient transgene-free genome editing for the autotetraploid cultivated alfalfa. Nat Commun.

[18] Shen C, Du H, Chen Z (2020). The chromosome-level genome sequence of the autotetraploid alfalfa and resequencing of core germplasms provide genomic resources for alfalfa research. Mol Plant.

[19] Vilar JMG, Saiz L (2013). Systems biophysics of gene expression. Biophys J.

[20] Chen S, Calvo JM (2002). Leucine-induced dissociation of Escherichia coli Lrp hexadecamers to octamers. J Mol Biol.

[21] Vimala Y, Lavania S, Lavania UC (2021). Chromosome change and karyotype differentiation–implications in speciation and plant systematics. Nucleus.

[22] Wu S (2020). Genetic contribution of paleopolyploidy to adaptive evolution in angiosperms. Mol Plant.

[23] Dröge-Laser W (2018). The *Arabidopsis* bZIP transcription factor family-an update. Curr Opin Plant Biol.

[24] Xu G, Guo C, Shan H, Kong H (2012). Divergence of duplicate genes in exon-intron structure. Proc Natl Acad Sci U S A.

[25] Friedman R, Hughes AL (2001). Gene duplication and the structure of eukaryotic genomes. Genome Res.

[26] Hu W, Wang Y, Bowers C, Ma H (2003). Isolation, sequence analysis, and expression studies of florally expressed cDNAs in *Arabidopsis*. Plant Mol Biol.

[27] Barth O, Vogt S, Uhlemann R, Zschiesche W, Humbeck K (2009). Stress induced and nuclear localized HIPP26 from *Arabidopsis thaliana* interacts via its heavy metal associated domain with the drought stress related zinc finger transcription factor ATHB29. Plant Mol Biol.

[28] Knight CA, Vogel H, Kroymann J, Shumate A, Witsenboer H, Mitchell-Olds T (2006). Expression profiling and local adaptation of Boechera holboellii populations for water use efficiency across a naturally occurring water stress gradient. Mol Ecol.

[29] Chen C, Chen H, Zhang Y (2020). TBtools: an integrative toolkit developed for interactive analyses of big biological data. Mol Plant.

[30] Li H, Handsaker B, Wysoker A (2009). The sequence alignment/map format and SAMtools. Bioinformatics.

[31] Trapnell C, Roberts A, Goff L (2012). Differential gene and transcript expression analysis of RNA-seq experiments with TopHat and Cufflinks. Nat Protoc.

[32] Gentleman RC, Carey VJ, Bates DM (2004). Bioconductor: open software development for computational biology and bioinformatics. Genome Biol.

